# Oncolytic Newcastle disease virus expressing chimeric antibody enhanced anti-tumor efficacy in orthotopic hepatoma-bearing mice

**DOI:** 10.1186/s13046-015-0271-1

**Published:** 2015-12-21

**Authors:** Ding Wei, Qian Li, Xi-Long Wang, Yuan Wang, Jing Xu, Fei Feng, Gang Nan, Bin Wang, Can Li, Ting Guo, Zhi-Nan Chen, Huijie Bian

**Affiliations:** State Key Laboratory of Cancer Biology, Cell Engineering Research Center and Department of Cell Biology, Fourth Military Medical University, No. 169, Changle West Road, Xi’an, 710032 China

**Keywords:** Newcastle disease virus, Oncolytic virotherapy, Hepatocellular carcinoma, cHAb18, CD147

## Abstract

**Background:**

Oncolytic virus which arms the therapeutic gene to enhance anti-tumor activity is a prevalent strategy to improve oncovirotherapy of cancer. Newcastle disease virus (NDV) is a naturally oncolytic virus used for cancer therapy. Previously, we generated a mouse-human chimeric HAb18 antibody (cHAb18) against tumor-associated antigen CD147 and demonstrated the inhibition of invasion and migration of hepatocellular carcinoma (HCC) cells. Here, we constructed a recombinant NDV carrying intact cHAb18 gene (rNDV-18HL) based on Italien strain using a reverse genetics system.

**Method:**

Recombinant rNDV-18HL was generated using reverse genetics technology. The characteristics of virally expressed cHAb18 antibody were identified by western blot, enzyme-linked immunosorbent assay, transwell invasion assay, and surface plasmon resonance technology. The biodistribution of recombinant rNDV-18HL using orthotopic xenograft mouse model was assessed with living imaging and immunohistochemistry. Kaplan-Meier survival curves and the log-rank test were performed to analyze the anti-tumor activity of rNDV-18HL.

**Results:**

The cHAb18 was produced in rNDV-18HL-infected cells followed by releasing into the supernatant by cytolysis. The rNDV-18HL-encoded cHAb18 antibody kept affinity for CD147 and showed inhibiting the migration and invasion of HCC cells. Viral replication and virulence were not attenuated by the incorporation of cHAb18 gene which significantly enhanced the suppression of relict tumor cell migration. The rNDV-18HL selectively replicated in orthotopic HCC xenografts leading to cHAb18 expression *in situ*, which induced the tumor necrosis, reduced the intrahepatic metastasis, and prolonged the survival in mice.

**Conclusions:**

This study provides a new strategy of arming oncolytic NDV with therapeutic antibody to enhance anti-tumor efficacy of cancer therapy.

## Background

Oncolytic virus (OV) has the special property with selective infection of tumor cells, leading to oncolysis of cancer cells and minimal toxicity to normal tissues, which is administrated in cancer therapy called oncolytic virotherapy. A great variety of OVs, including genetically engineered and natural viruses have shown promise in preclinical models and clinical studies [1].

NDV is a member of the *Paramyxoviridae* with a negative non-segment single strand RNA genome which encodes six proteins including nucleocapsid protein (NP), phosphoprotein (P), matrix protein (M), fusion protein (F), haemagglutinin-neuraminidase (HN), and RNA dependent RNA polymerase (L). NDV strains are classified into velogenic (highly virulent), mesogenic (intermediate virulence), and lentogenic (nonvirulent) based on the virulence in the natural host [2]. The virus is regarded as a natural OV for solid tumor therapy in clinic trials and shows minimal side-effects with systemic administration [3, 4]. Recently, the toxicity, biodistribution, and shedding of NDV in non-human primates under intravenous injection were evaluated, demonstrating the safety for intravenous administration [5].

In a previous study, we identified NDV Italien strain belonged to the velogenic strain [6]. By reverse genetics technology, we demonstrated that NDV Italien was able to carry exogenous genes without affecting virus replication [7]. The results suggest that NDV Italien can be served as a candidate vector carrying therapeutic transgenes to enhance the therapeutic indices for armed oncolytic virotherapy of cancers.

Several recombinant OVs, such as herpes simplex virus (HSV) [8, 9], vaccinia virus [10], and vesicular stomatitis virus [11] are armed with granulocyte-macrophage colony-stimulating factor (GM-CSF) to enhance systemic anti-tumor immune response. OncoVEX^GM-CSF^, a recombinant HSV expressing GM-CSF in phase III trial for treatment of melanoma, was proved to eliminate cancer cells by inducing local and systemic antigen-specific T cell responses and decreasing suppressive immune cell populations [12]. In October 2015, the US Food and Drug Administration approved the injectable formulation of OncoVEX^GM-CSF^, with the brand name Imlygic, for the treatment of melanoma in patients with inoperable tumors.

Therapeutic antibodies have achieved considerable success in treating patients with haematological malignancies and solid tumors. The mechanisms of tumor cell killing by antibodies are summarized as the direct action of the antibody, payload delivery, and specific effects of an antibody on the tumor vasculature and stroma. Intact antibody can also trigger antibody-dependent cellular cytotoxicity and complement-dependent cytotoxicity, which improve the antitumor therapeutic effect greatly. Several monoclonal antibody drugs, such as trastuzumab, bevacizumab, and DTA-1 are used in the combination therapy with OVs to enhance the antitumor efficacy in recent years [13–15]. Another strategy is to construct recombinant OVs which express antibody as an effector to augment the cytotoxicity of OVs. A recombinant oncolytic adenovirus expressing anti-CTLA4 antibody was generated and showed an effective antitumor activity in vivo [16].

Previously we developed a murine monoclonal antibody, HAb18 (generic named metuximab) targeting CD147 molecule. CD147 is over-expressed in HCC cells and involved in tumor cell invasion [17] and closely related to prognosis in patients with HCC [18–22]. Iodine [^131^I] metuximab injection was approved in China for treatment of HCC in 2005. It has been proved to have a beneficial treatment effect on prevention of tumor recurrence in patients with HCC [23, 24]. We generated a mouse-human chimeric cHAb18 antibody that derived from murine HAb18 and showed inhibition of HCC cell invasion and migration [25].

In order to combine the direct oncolysis induced by NDV with the antibody-targeted therapy, we here constructed a recombinant NDV Italien carrying a chimeric cHAb18 gene which expressed intact antibody in tumor tissue along with viral replication. The results showed that the recombinant NDV inhibited the intrahepatic metastasis of HCC and prolonged the survival in orthotopic hepatoma-bearing mice.

## Methods

### Cell lines and viruses

The BSR-T7/5 cell line was kindly donated by Prof. Karl-Klaus Conzelmann (Ludwig Maximilian University, Munich, Germany). SMMC-7721, HepG2, HuH-7, and BHK-21 cell lines were purchased from the Cell Bank of the Chinese Academy of Sciences (Shanghai, China). All cells were grown in Dulbecco’s Modified Eagle Medium (GE healthcare, Little Chalfont, UK) with 10 % fetal bovine serum and maintained at 37 °C in a humidified incubator supplied with 5 % CO_2_.

The NDV Italien strain was obtained from Prof. Volker Schirrmacher (German Cancer Research Center, Heidelberg, Germany) and prepared as reported previously [6]. The recombinant rNDV-Luci which expressed firefly luciferase was generated based on Italien strain in our previous works [7].

### Generation of recombinant rNDV-18HL

**Fig. 1:**
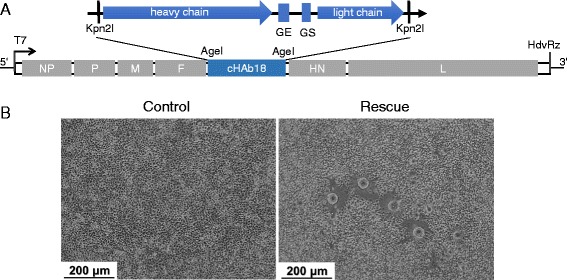
Construction and rescue of rNDV-18HL. **a** Schematic diagram of the rNDV-18HL genome which contained the genes of light and heavy chains of human-mouse chimeric antibody cHAb18. Two transcriptional cassettes containing light and heavy chains with gene start (GS) and gene end (GE) signals were inserted into the AgeI (isoaudamers of Kpn2I) site between F and HN genes. **b** Syncytia cells induced by recombinant rNDV-18HL which was rescued by co-transfection of pBR-rNDV-18HL with three helper plasmids into BSR-T7/5 cells. BSR-T7/5 cells co-transfected with three helper plasmids were served as control. Scale bar = 200 μm

The gene of chimeric antibody cHAb18 was cloned from the plasmid 18HL-pDHL which was constructed in our previous study [25]. The light and heavy chains were cloned respectively using primer pairs 18 L-F/R and 18H-F/R (Table 1) and then joined by overlap extension PCR. The annealed fragment was provided with an extra gene start (GS) and gene end (GE) sequences and flanked on either side with a Kpn2I restriction site as shown in Fig. 1a. The PCR products were purified with a gel extraction kit (Omega Bio-Tek, Norcross, NA) and cloned into pBR-rNDV which was constructed based on the NDV Italien in our previous study [7] using the AgeI restriction site. The resulting plasmid was named pBR-rNDV-18HL.

**Table 1:** Primers for amplification of cHAb18 and identification of rNDV-18HL^a^

Primer names	Sequences (5' - 3')
18 L-F	TTAGAAAAAAATACGGGTAGAAATAAGCCACCATGGACTCACATACTC
18 L-R	TATTCCGGATTAACACTCTCCCCTGTTGAA
18H-F	ATATCCGGAGCCACCATGAACTTCGGGCTGAGC
18H-R	TTCTACCCGTATTTTTTTCTAAGTGATAGTGATCATTTACCCGGAGAC
VT-F	GACGGGATAACTCTGAGG
VT-R	GTATGCCAACAAGGTCGC

^a^Underline indicated the overlap regions in overlap PCR

The recombinant NDV carrying cHAb18 antibody (rNDV-18HL) was rescued using a standard protocol as described previously [7]. Briefly, 2.5 μg of pBR-rNDV-18HL, 1 μg of T7-NP, 1 μg of T7-P, and 0.5 μg of T7-L were mixed and co-transfected into BSR-T7/5 cells using Lipofectamine 2000 (Life Technologies, Carlsbad, CA). Culture supernatant was collected at 3 days post-transfection and the recombinant rNDV-18HL was purified using plaque assay.

To identify recombinant rNDV-18HL, the genome RNA of virus was extracted using MiniBEST viral RNA/DNA extraction kit (TaKaRa Bio, Otsu, Japan) and the foreign fragment between the HN and F genes were amplified using primers VT-F and VT-R (Table 1). Then PCR product was purified and sequencing for verification.

### Identification of virally expressed cHAb18 antibody

To identify rNDV-18HL-expressed cHAb18 antibody, BHK-21 cells were infected with rNDV-18HL at MOI = 0.01. The culture supernatant was collected at 36 h post-infection and purified using Protein G affinity chromatography column (Pharmacia Inc., Peapack, USA). The cHAb18 was detected using western blot analysis under reduced condition, which following the standard protocol using anti-human IgG antibody (Fab specific) (Sigma, Brooklyn, USA).

To quantitate the cHAb18 production at different time points, BHK-21 cells were infected with rNDV-18HL at MOI = 0.01 and the culture supernatants were collected at different time of post-infection. The concentration of cHAb18 was detected using indirect ELISA assay. Briefly, the culture supernatants and standards were added to the ELISA wells which were coated with CD147 extracellular domain we produced previously [26]. After the incubation, anti-human IgG antibody labeled with horseradish peroxidase (Sigma, Brooklyn, USA) was added and the concentration was measured using Epoch microplate spectrophotometer (BioTek, Winooski, VT).

### Measurement of cHAb18 affinity for CD147

To determine the antibody affinity, cHAb18 was purified from culture supernatant using Protein G affinity chromatography column (Pharmacia Inc, Peapack-Gladstone, NJ). The equilibrium dissociation constant (K_D_) of cHAb18 was measured by surface plasmon resonance (SPR) technology with ProteOn™ GLC sensor (Bio-Rad Inc, Hercules, CA) according to the procedure described previously [25]. The data were analyzed using a 1:1 Langmuir binding model with global fitting (BIAevaluation software, BIAcore, Uppsala, Sweden).

### Viral proliferation

To investigate the proliferation of rNDV-18HL, BHK-21 cells in a six-well-plate were infected with NDV (MOI = 0.01). The culture supernatant was collected at different time of post-infection and the viral titer was measured by end-point dilution assay (TCID_50_/ml). Using BHK-21 cells with eight replicates for each dilution, the TCID_50_ per milliliter was calculated according to the method of Reed and Muench [27] and each assay was repeated three times.

### Cell viability

Three human HCC cell lines, HepG2, SMMC-7721, and HuH-7 were infected with NDV at MOI = 0.01. The viability of cells at different time points was assessed by MTT cell proliferation and cytotoxicity assay kit (Beyotime Institute of Biotechnology, Haimen, China) with three repetitions for each sample. Absorbance at 570 nm was determined with BIO-TEK microplate readers (BioTek Instruments, Winooski, VT).

### Transwell invasion and wound healing assays

The inhibition of cell invasion and migration by cHAb18 derived from rNDV-18HL was performed in SMMC-7721 cells using transwell invasion and wound healing assays according to the procedures described previously [25]. To detect the synergistic effect of rNDV-18HL on repression of cell migration, 2 × 10^4^ SMMC-7721 cells were cultured in a transwell chamber with 8 μm pore (Millipore, Billerica, MA) and infected with rNDV-18HL at MOI = 0.01. At 3 days post-infection, cells attached to the lower side of membrane were wiped with sterile cotton swabs. The medium in the upper chamber was replaced with fresh serum-free medium. The next day cells re-attached on the lower side were fixed with 90 % ethanol for 3 min and stained with 0.1 % crystal violet for 10 min. The stained areas were measured using ImageJ software (National Institutes of Health, Bethesda, MD).

### Orthotopic xenograft model of human hepatoma and oncolytic virotherapy

The 6 to 8-week-old female BALB/c nude mice were purchased from Animal Resources Centre (Shanghai, China). All mice were housed at a constant temperature (24 °C) in pathogen-free conditions with a 12:12 h light–dark photoperiod, and allowed food and water *ad libitum*. The protocols involving animals were conducted according to the Animal Welfare Act and approved by the Animal Care and Use Committee of Fourth Military Medical University (No. 2012065). The surgery was performed under sodium pentobarbital anesthesia, and all efforts were made to minimize animal suffering. The 5 × 10^6^ SMMC-7721 cells in 100 μl matrigel (BD Bioscience) were implanted into hepatic lobule of mice. Mice were randomized into four groups in one week after implantation, which were received tail intravenous injection twice weekly for 3 weeks and denoted as saline (0.9 % NaCl), cHAb18-treated (2 mg/kg/injection), NDV Italien-treated (5 × 10^7^ PFU/injection), and rNDV-18HL-treated (5 × 10^7^ PFU/injection) groups. Body weight was measured every three days since implantation. The endpoint of observation for each mouse was determined by the following conditions: lack of responsiveness to manual stimulation, immobility, and/or inability to eat or drink. The mice meeting one of the criteria were euthanized using pentobarbital.

### Living imaging of mice

Mice were infected with 5 × 10^7^ PFU rNDV-Luci by tail intravenous injection every two days for three times. At 24 h post-final injection, mice were anesthetized with isoflurane mixed with oxygen and injected intraperitoneally with D-luciferin potassium salt (1.5 mg/mouse) for 10 min before imaging. Bioluminescence images were captured using Xenogen IVIS spectrum in vivo imaging system (Caliper Life Sciences, Hopkinton, MA).

### Immunohistochemistry

Mice were sacrificed and organs were fixed with 10 % formalin followed by embedding in paraffin. Tissue sections (4 μm) were obtained from the paraffin blocks, stained with hematoxylin and eosin (H&E) and were subjected to immunohistochemistry by staining with rabbit anti-NDV HN protein antibody (Bioss, San Diego, CA), rabbit anti-human IgG Fc, or rabbit anti-Ki-67 (Abcam, Cambridge, UK) using a streptavidin-peroxidase staining kit (ZSGB-Bio., Beijing, China).

### Statistical analysis

Statistical data were obtained with SigmaPlot (Systat Software, San Jose, CA). One-way analysis of variance (ANOVA) was used to determine significance between groups followed by the Dunnett’s test to analyze the differences between treated group and control group. A two-tailed Student *t* test was applied for comparison of two individual data. Survival analysis was determined by Kaplan-Meier estimation and log-rank test. A *P*-value less than 0.05 is considered statistically significant. Data were presented as means plus the standard deviation (SD).

## Results

### Generation of recombinant NDV carrying cHAb18 antibody

The genes of light and heavy chains of cHAb18 were introduced in the non-coding region between F and HN to generate a recombinant plasmid pBR-rNDV-18HL (Fig. 1a). The pBR-rNDV-18HL and the three helper plasmids (T7-NP, T7-P, and T7-L) [28] were co-transfected into BSR-T7/5 cells. At 3 days post-transfection, plaques were observed and the recombinant NDV carrying cHAb18 (rNDV-18HL) was identified by sequencing non-coding region between HN and F genes (Fig. 1b).

**Fig. 2:**
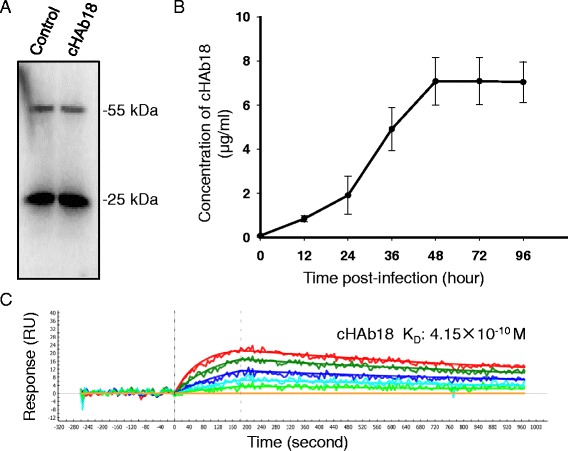
Identification of rNDV-18HL. **a** BHK-21 cells were infected with rNDV-18HL and the human IgG was purified from the supernatant for detecting cHAb18 expression using western blot analysis. The purified cHAb18 antibody from stably transfected CHO clone was served as control. **b** The level of cHAb18 antibody was determined by ELISA in culture supernatants of rNDV-18HL-infected BHK-21 cells at different time of post-infection (*n* = 5). **c** One-shot kinetics for the interaction between virally expressed cHAb18 antibody and CD147 antigen was detected with surface plasmon resonance technology. Each set of five sensograms displayed the responses to the five diluted concentrations of recombinant CD147 interacting with one immobilization level of cHAb18 antibody. RU was denoted as resonance unit

We detected the antibody level in the supernatant of virus-infected BHK-21 cells. Western blot assay showed an expression of IgG with 25 kDa and 55 kDa bands under the reduced condition, which indicated the light and heavy chains of antibody, respectively (Fig. 2a). Sandwich ELISA showed that cHAb18 antibody could bind the CD147 antigen and anti-human IgG antibody simultaneously. The concentration of cHAb18 was gradually increased with the time of viral infection which reached the peak 7.07 μg/ml at 48 h post-infection and then kept the platform (Fig. 2b). SPR analysis showed that the K_D_ of cHAb18 was 4.15 × 10^−10^ M, which was very close to the same antibody produced from genetically engineered CHO cells [25] (Fig. 2c). All these results showed that the replication of rNDV-18HL was accompanied by an accelerated expression of intact cHAb18 antibody.

### rNDV-18HL kept the oncolytic features as wild strain NDV Italien

**Fig. 3:**
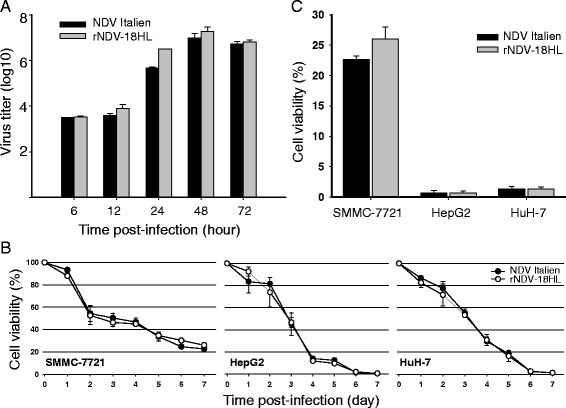
Characteristics of rNDV-18HL. **a** Proliferation dynamic of rNDV-18HL. BHK-21 cells were infected with either rNDV-18HL or NDV Italien at MOI = 0.01. At 6, 12, 24, 36, 48, and 72 h post-infection, culture supernatants were collected and the viral titer was determined (TCID_50_/ml) (*n* = 3). **b** Cytotoxicity assay of rNDV-18HL. SMMC-7721, HuH-7, and HuH-7 cells were infected with either rNDV-18HL or NDV Italien for 7 days. The cell viability was measured using MTT assay (*n* = 5). The cell viability of the three HCC cell lines at 7 days post-infection was shown in (**c**)

We analyzed the proliferation of rNDV-18HL in BHK-21 cells. As shown in Fig. 3a, no significant differences of virus titer between rNDV-18HL and NDV Italien were detected, which indicated that the insertion of cHAb18 gene did not disturb the replication capacity of NDV and the recombinant rNDV-18HL still kept the same virulence as the wild-type strain.

Three human HCC cell lines, HepG2, HuH-7, and SMMC-7721 were infected with either rNDV-18HL or NDV Italien, and the cell viability was measured by MTT assay each day during the first week post-inoculation. As shown in Fig. 3b, rNDV-18HL induced the cytolysis effects with the similar features by NDV Italien in all cell lines. HepG2 and HuH-7 cells were more sensitive to both rNDV-18HL and NDV Italien replication and almost dead at 7 days post-infection compared with SMMC-7721 cells (Fig. 3c). These results indicated that rNDV-18HL retained its oncolytic property with diverse sensitivity to different tumor cell lines.

### rNDV-18HL enhanced the inhibition of tumor cell migration and invasion

**Fig. 4:**
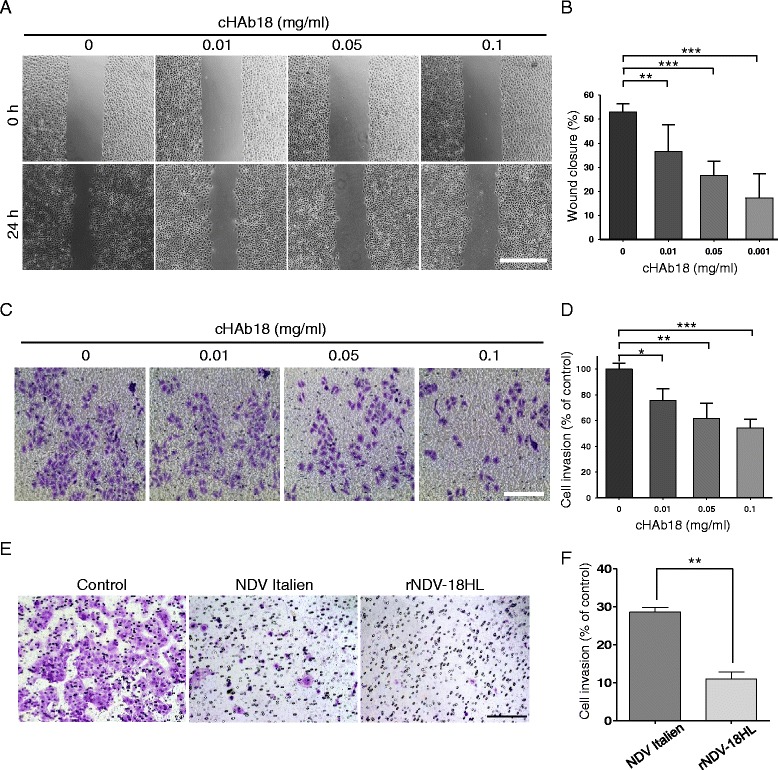
The inhibition of HCC cell motility by virally expressed cHAb18 and rNDV-18HL. **a** Wound healing assay detected the migration of SMMC-7721 cells which were treated with virally expressed cHAb18 for 24 h. **b** The relative wound closure was measured using ImageJ (*n* = 5). Scale bar = 500 μm. **c** SMMC-7721 cells in transwell chamber coated with matrigel were treated with virally expressed cHAb18 for 24 h. The invaded cells were stained with crystal violet. Scale bar = 200 μm. **d** The invaded cells were quantified by determining the absorption at 570 nm (*n* = 3). **e** Transwell migration assay detected the migration of SMMC-7721 cells which were treated with either rNDV-18HL or NDV Italien. The cells on the lower side of membrane were stained with crystal violet. Scale bar = 200 μm. **f** The stained area of each image was measured using ImageJ (*n* = 5). * *P* < 0.05, ** *P* < 0.01, *** *P* < 0.001

The CHO cells-produced cHAb18 had been proved to inhibit tumor cell invasion and migration in our previous study [25]. Here, we verified that rNDV-18HL-expressed cHAb18 antibody still suppressed the migration and invasion of SMMC-7721 cells in dose-dependent manners, as detected by wound healing (Fig. 4a and b) and transwell invasion assays (Fig. 4c and d). Furthermore, using transwell chamber, we evaluated the anti-migration ability of rNDV-18HL. At 4 days post-infection, most cells were destroyed by rNDV-18HL or NDV Italien directly, meanwhile, the migration of relict cells were inhibited significantly in rNDV-18HL-treated cells compared with that of NDV Italien (Fig. 4e and f). Because rNDV-18HL and NDV Italien had the same oncolytic property as demonstrated in Fig. 3, we believed that the anti-motility effect was caused by the expression of cHAb18 antibody.

### rNDV-18HL specifically replicated in orthotopic HCC xenografts leading to cHAb18 antibody expression *in situ* and tumor central necrosis

**Fig. 5:**
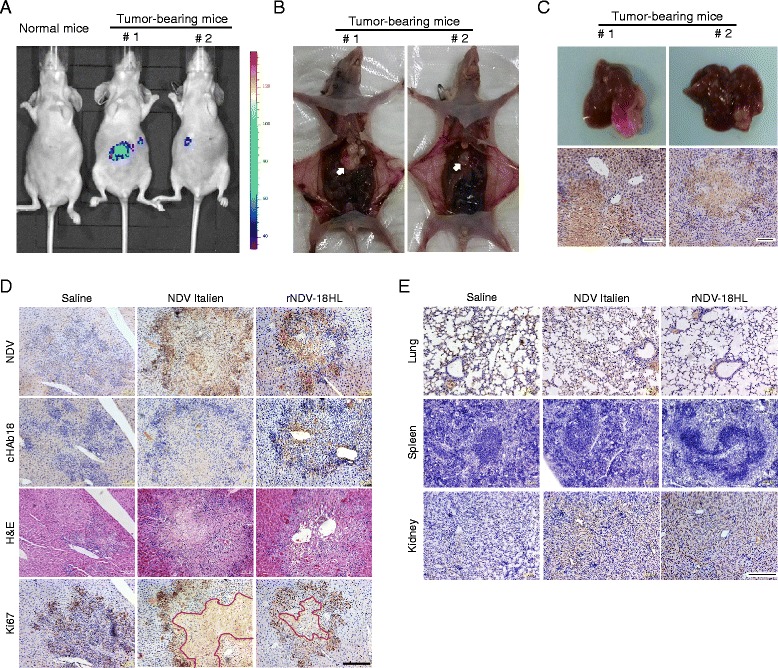
Tumor-selective replication of rNDV-18HL in orthotopic HCC xenograft mice induced tumor necrosis and cHAb18 antibody expression *in situ*. **a** Orthotopic HCC xenograft nude mice were treated intravenously with rNDV-Luci. At 24 h post-injection, the mice were anesthetized and visualized by luminescence using Xenogen IVIS spectrum. The exposure time was 1 min, and the images were pseudocolored according to the scale on the right. **b** The mice were sacrificed and dissected to identify the location of tumors in the livers (*arrow*). **c** Tumor-bearing livers were excised (*top*) and the tissue sections were stained with rabbit anti-NDV HN protein antibody to indicate the distribution of NDV (bottom). Scale bar = 100 μm (**d**) Orthotopic HCC xenograft nude mice were treated intravenously with either rNDV-18HL or NDV Italien twice weekly for 3 weeks. The mice were sacrificed and the tumor-bearing livers were excised. The consecutive tissue sections were used for visualizing tumor loci by H&E staining, and detecting NDV, cHAb18 antibody, and Ki-67 expression by immunohistochemistry. The tumor tissue necrosis was indicated by red line. Scale bar = 200 μm. **e** Normal organs were also excised and stained with anti-NDV HN monoclonal antibody to detect the distribution of NDV. Scale bar = 200 μm

To evaluate the selective replication of rNDV-18HL in tumors, a model of human HCC xenograft in athymic nude mice was established by orthotopic liver transplantation. The recombinant rNDV-Luci was injected into the mice via tail vein. At 3 days post-infection, luminescence was detected in the epigastric regions in tumor-bearing mice using in vivo imaging system (Fig. 5a). Autopsy findings indicated the tumor formation in livers, and it seemed that the larger tumor loci (#1) had a stronger luminescence intensity than the smaller one (#2) (Fig. 5a and b). Immunohistochemistry assay detected that the NDV distribution was limited in the tumors (Fig. 5c).

Next, the orthotopic HCC xenograft athymic mice were intravenously treated with rNDV-18HL and sacrificed at 24 h post-injection. Using consecutive tissue sections, immunohistochemistry analysis showed that both NDV and cHAb18 expression were only presented in the tumor tissues of mice injected with rNDV-18HL (Fig. 5d), while the virus was spare in normal organs (Fig. 5e). The replication of rNDV-18HL in tumor not only facilitated antibody expression, but also induced tumor tissue necrosis as detected by H&E staining and anti-Ki-67 staining. Antigen Ki-67, a cellular marker for proliferation, is present during all active phases of the cell cycle, but is absent from resting cells. In our study, the Ki-67-positive cells were mainly observed in the tumor margin in both virus-treated groups with proliferating and hematoxylin-positive cells distributed sporadically. Whereas, in saline group, Ki-67-positive cells were displayed on tumor margin and center region as well as. No necrosis was observed (Fig. 5d). These results suggest that recombinant NDV Italien infected tumor tissue effectively and replicated in tumor cells selectively leading to the exogenous antibody gene expression and tumor central necrosis.

### rNDV-18HL reduced the intrahepatic metastasis and prolonged the survival in mice

**Fig. 6:**
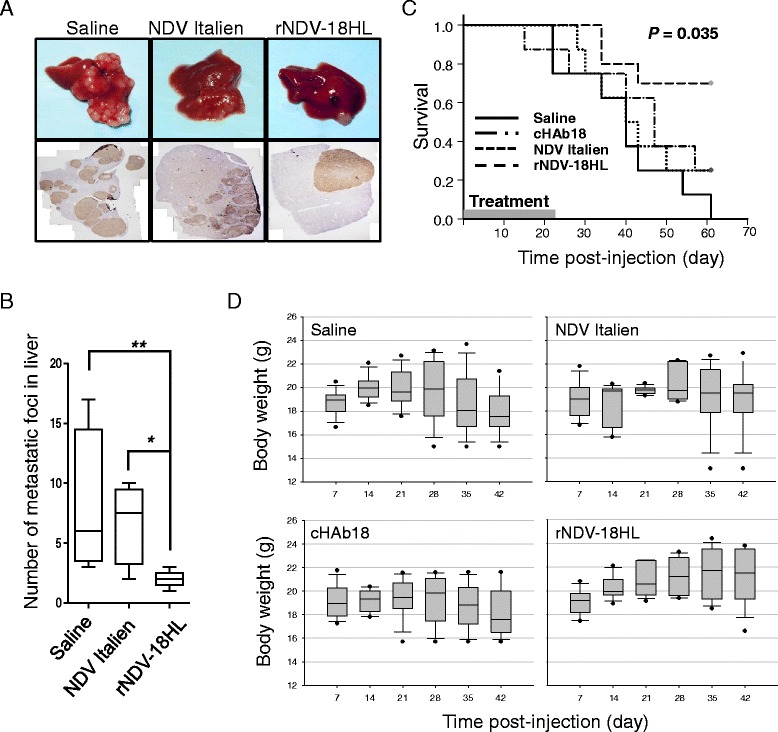
Enhanced anti-tumor effect of rNDV-18HL. **a** Orthotopic transplantation HCC nude mice were treated with rNDV-18HL, NDV Italien, or normal saline through tail intravenous injection twice weekly for 3 weeks. At 24 h post-injection, three mice of each group were sacrificed to investigate the metastatic foci in the liver. The representative images were shown by gross observation (*upper*) and Ki-67 staining (*bottom*). **b** The number of metastatic foci was counted (three Ki-67 staining slides were selected randomly for each sample, and only separated metastatic foci bigger than 50 μm was counted) and indicated as means ± SD. * *P* < 0.05, ** *P* < 0.01. **c** Orthotopic transplantation HCC nude mice were treated with rNDV-18HL, cHAb18, NDV Italien, or normal saline through tail intravenous injection twice weekly for 3 weeks. The number of mice in each group was 11. The treated mice were monitored daily and the Kaplan-Meier survival curve was plotted and analyzed by log-rank test. **d** The body weights were recorded every 3 days after treatment

To investigate the synergistic effects of rNDV-18HL on anti-tumor activity, intrahepatic metastatic foci were checked. As shown in Fig. 6a and b, mice treated with rNDV-18HL at 24 h post-treatment had less metastatic foci compared with NDV Italien (*P* < 0.05) or saline (*P* < 0.01), while no differences of metastatic foci between NDV Italien and saline groups were observed.

The survival curves and log rank test showed that rNDV-18HL treatment significantly prolonged the survival in mice (*P* = 0.035) (Fig. 6c). Meanwhile, there was no significant difference of survival among NDV Italien, cHAb18, and saline groups although beneficial trends were detected in cHAb18 and NDV Italien-treated groups, which was similar to our previous studies [7, 25]. The results suggested that oncolytic NDV carrying cHAb18 antibody improved therapeutic effects by inhibiting tumor local metastasis contributing to the survival. The body weights of rNDV-18HL-treated group showed continuous increase during the period of observation, and NDV Italien-treated group was stable, while the cHAb18-treated and saline groups displayed downtrends after 26 days post-treatment (Fig. 6d). As the body weight loss could be caused by tumor progression and treatment-induced side-effects, our data revealed a benefit of rNDV-18HL treatment and less side-effect.

## Discussion

The “armed” OVs which combine gene therapy, immunotherapy, and oncovirotherapy are regarded as potential and promising agents for tumor therapy. Cytokines and therapeutic genes are desirable exogenous genes to enhance anti-tumor activity of OVs [29–31]. Several genes, such as interferon [32], tumor necrosis factor-related apoptosis inducing ligand (TRAIL) [33], interleukin-7 (IL-7) [34], cytosine deaminase [35], IL-2 [33, 36], GM-CSF [37], and IL-15 [38] have been reported to be incorporated into lentogenic strains of NDV genome. A proof-of-principle study demonstrates the transgenic expression of a full IgG antibody recognizing ED-B type III of fibronectin by mesogenic NDV strain MTH68 [39]. Here, we constructed a recombinant velogenic NDV strain Italien carrying humanized therapeutic antibody to enhance the therapeutic effect of tumor therapy. We introduced the cHAb18 antibody between the F and HN genes using two independent transcriptional cassettes to express heavy and light chains of antibody. The insertion of antibody gene into this site struck a balance between the expression level of exogenous gene and the viral proliferation. It is reported that administration of antibodies carries the risk of some adverse effects, including infusion reaction, cardio toxicity, and hypersensitivity, which can cause a serious problem even death [40]. We supposed that the tumor-selective delivery of antibodies by oncolytic NDV limited the virus spreading into circulation system or normal organs, which might facilitate to diminish the side effects of antibody drug our previous study, it is proved that cHAb18 antibody directs against CD147 to inhibit matrix metalloproteinase-2/9 production and rearrange actin cytoskeleton via suppressing integrin-FAk-PI3K/Akt-girdin signaling pathway, thus suppressing cell invasion and motility [25]. Our study indicated that the recombinant rNDV-18HL combined the oncolytic virotherapy and antibody therapy which showed an increased antitumor activity than NDV Italien or cHAb18 antibody alone. On the one hand, rNDV-18HL infected tumor cells specifically and caused tumor lysis directly. On the other hand, virally expressed cHAb18 which was released from the lysate of tumor cells inhibited the metastasis of tumor cells.

## Conclusions

The recombinant NDV carrying the chimeric cHAb18 monoclonal antibody improves the therapeutic effect and shows a new strategy for tumor therapy.

### Ethics approval and consent to participate

The protocols involving animals were conducted according to the Animal Welfare Act and approved by the Animal Care and Use Committee of Fourth Military Medical University (No. 2012065).

### Consent for publication

Not applicable.

## Abbreviations

NDV: Newcastle disease virus
OV: Oncolytic virus
GM-CSF: Granulocyte-macrophage colony-stimulating factor
SPR: Surface plasmon resonance
H&E: Hematoxylin and eosin
SD: Standard deviation
